# Prospective mapping of viral mutations that escape antibodies used to treat COVID-19

**DOI:** 10.1101/2020.11.30.405472

**Published:** 2020-12-01

**Authors:** Tyler N. Starr, Allison J. Greaney, Amin Addetia, William W. Hannon, Manish C. Choudhary, Adam S. Dingens, Jonathan Z. Li, Jesse D. Bloom

**Affiliations:** 1 Basic Sciences and Computational Biology, Fred Hutchinson Cancer Research Center, Seattle, WA 98109; 2 Department of Genome Sciences, University of Washington, Seattle, WA 98109; 3 Medical Scientist Training Program, University of Washington, Seattle, WA 98109; 4 Brigham and Women’s Hospital, Harvard Medical School, Boston, MA 02115; 5 Molecular and Cellular Biology Graduate Program, University of Washington, Seattle, WA 98109; 6 Howard Hughes Medical Institute, Seattle, WA 98109

## Abstract

Antibodies are becoming a frontline therapy for SARS-CoV-2, but the risk of viral evolutionary escape remains unclear. Here we map how all mutations to SARS-CoV-2’s receptor-binding domain (RBD) affect binding by the antibodies in Regeneron’s REGN-COV2 cocktail and Eli Lilly’s LY-CoV016. These complete maps uncover a single amino-acid mutation that fully escapes the REGN-COV2 cocktail, which consists of two antibodies targeting distinct structural epitopes. The maps also identify viral mutations that are selected in a persistently infected patient treated with REGN-COV2, as well as in lab viral escape selections. Finally, the maps reveal that mutations escaping each individual antibody are already present in circulating SARS-CoV-2 strains. Overall, these complete escape maps enable immediate interpretation of the consequences of mutations observed during viral surveillance.

Antibodies are being developed as therapeutics to combat SARS-CoV-2 (1). Antibodies against some other viruses can be rendered ineffective by viral mutations that are selected during treatment of infected patients (2, 3) or that spread globally to confer resistance on entire viral clades (4). Therefore, determining *a priori* which SARS-CoV-2 mutations escape key antibodies is essential for assessing how mutations observed during viral surveillance impact the efficacy of antibody treatments.

Most leading anti-SARS-CoV-2 antibodies target the viral receptor-binding domain (RBD), which mediates binding to ACE2 receptor (5, 6). We recently developed a deep mutational scanning method to map how all mutations to the RBD affect its function and recognition by antiviral antibodies (7, 8). This method involves creating libraries of RBD mutants, expressing them on the surface of yeast, and using fluorescence-activated cell sorting and deep sequencing to quantify how each mutation affects RBD folding, ACE2 affinity, and antibody binding (Fig. S1A). Here we applied this method to map all RBD mutations that escape binding by recombinant forms of the two antibodies in Regeneron’s REGN-COV2 cocktail (REGN10933 and REGN10987) (9, 10), and Eli Lilly’s LY-CoV016 antibody (also known as CB6 or JS016) (11) (Fig. S1B). REGN-COV2 was recently granted an emergency use authorization for treatment of COVID-19 (12), while LY-CoV016 is currently in phase 2 clinical trials (13).

We completely mapped RBD mutations that escape binding by the three individual antibodies as well as the REGN10933 + REGN10987 cocktail (Fig. 1A,B and zoomable maps at https://jbloomlab.github.io/SARS-CoV-2-RBD_MAP_clinical_Abs/). REGN10933 and REGN10987 are escaped by largely non-overlapping sets of mutations in the RBD’s receptor-binding motif (Fig. 1A), consistent with structural work showing that these antibodies target distinct epitopes in this motif (9). But surprisingly, one mutation (E406W) strongly escapes the cocktail of both antibodies (Fig. 1A). The escape map for LY-CoV016 also reveals escape mutations at a number of different sites in the RBD (Fig. 1B). Although some escape mutations impair the RBD’s ability to bind ACE2 or be expressed in properly folded form, many come at little or no cost to these functional properties (colors in Fig. 1A,B and Fig. S2)—an unfortunate consequence of the mutational tolerance of the RBD (7).

To validate the antigenic effects of key mutations, we performed neutralization assays using spike-pseudotyped lentiviral particles, and found concordance between the escape maps and neutralization assays (Fig. 1C and Fig. S3). As expected from the maps for the REGN-COV2 antibodies, a mutation at site 486 escaped neutralization only by REGN10933, whereas mutations at sites 439 and 444 escaped neutralization only by REGN10987—and so none of these mutations escaped the cocktail. But E406W escaped both individual REGN-COV2 antibodies, and thus also strongly escaped the cocktail. The identification of E406W as a cocktail escape mutation demonstrates how complete maps provide information beyond other standard approaches: structural analyses and viral-escape selections led Regeneron to posit that no single amino-acid mutation could escape both antibodies in the cocktail (9, 10), but our complete maps show this is not true.

To explore how well our escape maps explain the evolution of virus under antibody selection, we first examined data from Regeneron’s viral escape-selection experiments in which spike-expressing VSV was grown in cell culture in the presence of REGN10933, REGN10987, or the cocktail (10). That work identified five escape mutations from REGN10933, two from REGN10987, and none from the cocktail (Fig. 2A). All five cell-culture-selected mutations were prominent among the single-nucleotide accessible mutations in our escape maps (Fig. 2B), demonstrating concordance between the escape maps and viral evolution under antibody pressure in cell culture. Notably, E406W is not accessible by a single-nucleotide change, which may explain why it was not identified by the Regeneron cocktail selections despite being relatively well tolerated for RBD folding and ACE2 affinity.

To determine if the escape maps could also inform analysis of viral evolution in infected humans, we examined deep sequencing data from a persistently infected immunocompromised patient who was treated with REGN-COV2 at day 145 after diagnosis with COVID-19 (14). The late timing of treatment allowed ample time for the patient’s viral population to accumulate genetic diversity. Administration of REGN-COV2 was followed by rapid changes in the frequencies of five amino-acid mutations in the RBD (Fig. 2C and Fig. S4). Our escape maps showed that three of these mutations escaped REGN10933, and one escaped REGN10987 (Fig.2B). Notably, the mutations did not all sweep to fixation after antibody treatment: instead, there were competing rises and falls (Fig. 2C). This pattern has been observed in the adaptive within-host evolution of other viruses (15, 16), and occurs because of genetic hitchhiking and competition among viral lineages. Both these forces are apparent in the persistently infected patient (Fig. 2C and Fig S4C): E484A (not an escape mutation in our maps) hitchhikes with F486I (which escapes REGN10933) after treatment, and the viral lineage carrying N440D and Q493K (which escape REGN10987 and REGN10933, respectively) competes first with the REGN10933 escape-mutant Y489H, and then with the E484A / F486I lineage and Q493K-alone lineage.

Importantly, three of the four escape mutations in the REGN-COV2-treated patient were not identified in Regeneron’s viral cell-culture selections (Fig. 2B), illustrating an advantage of complete maps. Viral selections are “incomplete” in the sense that they only identify whatever mutations are stochastically selected in that particular cell-culture experiment. In contrast, complete maps annotate all mutations, which could include mutations that arise for reasons unrelated to treatment but incidentally affect antibody binding.

Of course, viral evolution is shaped by functional constraints as well as pressure to evade antibodies. The mutations selected in cell culture and the patient consistently met the following criteria: they escaped antibody binding, were accessible via a single-nucleotide change, and imposed little or no cost on ACE2 affinity (as measured by prior deep mutational scanning (7); Fig. 2D, Fig. S5). Therefore, complete maps of how mutations affect key biochemical phenotypes of the RBD (e.g., ACE affinity and antibody binding) can be used to assess likely paths of viral evolution. A caveat is that over longer evolutionary timeframes, the space of tolerated mutations could shift due to epistatic interactions, as has been previously observed in viral immune and drug escape (17–19).

The complete maps enable us to assess what escape mutations are already present among circulating SARS-CoV-2. We examined all human-derived SARS-CoV-2 sequences available as of November 12, 2020, and found a substantial number of RBD mutations that escaped one or more of the antibodies (Fig. 3). However, the only escape mutations present in >0.1% of sequences were the REGN10933 escape-mutant Y453F (0.2% of sequences) (10) and the REGN10987 escape-mutant N439K (1.2% of sequences, has an effect on neutralization as shown in both Fig. 1C and (20)). Y453F is associated with independent mink-associated outbreaks in the Netherlands and Denmark (22, 23); notably the mink sequences themselves sometimes also contain other escape mutations such as F486L (21). N439K is prevalent in Europe, where it has comprised a large percentage of sequences from regions including Scotland and Ireland (20, 23).

To determine if the escape maps could be rationalized from the structural interfaces of the antibodies and RBD, we projected the maps onto crystal or cryo-EM structures (Fig. 4A; interactive versions at https://jbloomlab.github.io/SARS-CoV-2-RBD_MAP_clinical_Abs/). As might be expected, escape mutations generally occur in the antibody-RBD interface. However, structures alone are insufficient to predict which mutations mediate escape. For example, LY-CoV016 uses both its heavy and light chains to bind a wide epitope overlapping the ACE2-binding surface, but escape is dominated by mutations at RBD residues that contact the heavy chain CDRs (Figs. 4A, S6E–G). In contrast, escape from REGN10933 and REGN10987 mostly occurs at RBD residues that pack at the antibody heavy/light-chain interface (Fig. 4A, S6A–D). The E406W mutation that escapes the REGN-COV2 cocktail occurs at a residue not in contact with either antibody (Fig. 4A). So overall, mutations at RBD residues that contact antibody do not always mediate escape, and several prominent escape mutations occur at residues not in contact with antibody (Fig. 4B, S6D,G).

Overall, we have completely mapped mutations that escape some of the leading antibodies used to treat COVID-19. These maps demonstrate that prior characterization of escape mutations was incomplete: for instance, overlooking a single amino-acid mutation that escapes both antibodies in the REGN-COV2 cocktail, and failing to identify most mutations that arose in a persistently infected patient treated with the cocktail. Of course, our maps still do not answer the most pressing question: will SARS-CoV-2 evolve widespread resistance to these antibodies? While the presence of escape mutations in the patient treated with REGN-COV2 is ominous, other viruses that typically cause self-limiting acute infections undergo extensive within-patient evolution only in long infections of immunocompromised patients (15) and not in the broader population (24). However, it is concerning that so many escape mutations impose little cost on RBD folding or receptor affinity, and that some of these mutations are already present at low levels among circulating viruses. Ultimately, it will be necessary to wait and see what mutations spread as SARS-CoV-2 circulates in the human population. Our work will help with the “seeing,” by enabling immediate interpretation of the effects of the mutations catalogued by viral genomic surveillance.

## Supplementary Material

Supplement 1

1

## Figures and Tables

**Figure 1.**
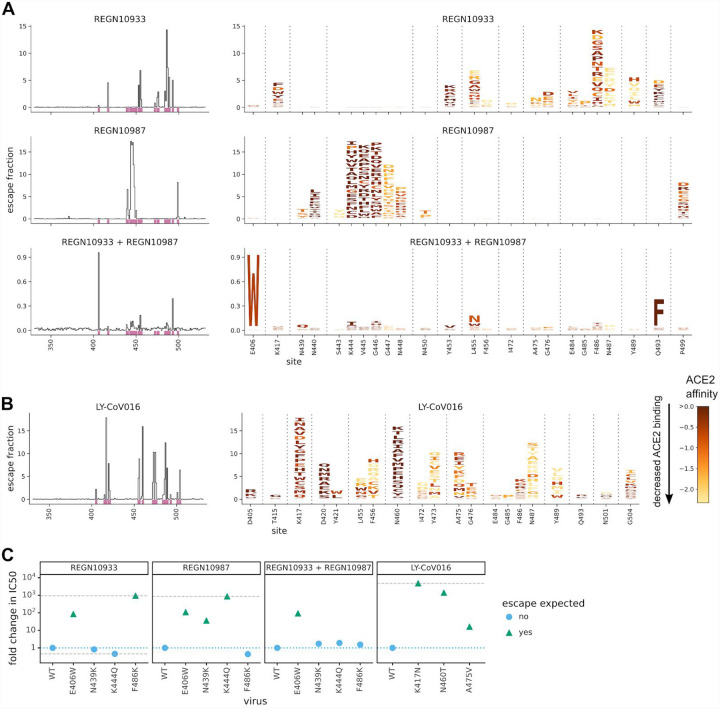
Complete maps of escape mutations from the REGN-COV2 antibodies and Ly-CoV016. (A) Maps for antibodies in REGN-COV2. Line plots at left show total escape at each site in the RBD. Sites of strong escape (purple underlines) are shown in logo plots at right. The height of each letter is proportional to how strongly that amino-acid mutation mediates escape, with a per-mutation “escape fraction” of 1 corresponding to complete escape. The y-axis scale is different for each row, so for instance E406W escapes all REGN antibodies but it is most visible for the cocktail as it is swamped out by other sites of escape for the individual antibodies. See https://jbloomlab.github.io/SARS-CoV-2-RBD_MAP_clinical_Abs/ for zoomable versions. Letters are colored by how mutations affect the RBD’s affinity for ACE2 (7), with yellow indicating poor affinity and brown indicating good affinity; see Fig. S2 for maps colored by how mutations affect expression of folded RBD. (B) Map for LY-CoV016. (C) Validation of key mutations in neutralization assays using pseudotyped lentiviral particles. Each point indicates the fold-increase in inhibitory concentration 50% (IC50) for a mutation relative to the unmutated “wildtype” (WT) Wuhan-Hu-1 RBD. The dotted blue line indicates wildtype-like neutralization sensitivity, and the dashed gray lines indicate upper and lower bounds on detectable fold changes. Point shapes / colors indicate if escape was expected at that site from the maps. Full neutralization curves are in Fig. S3.

**Figure 2.**
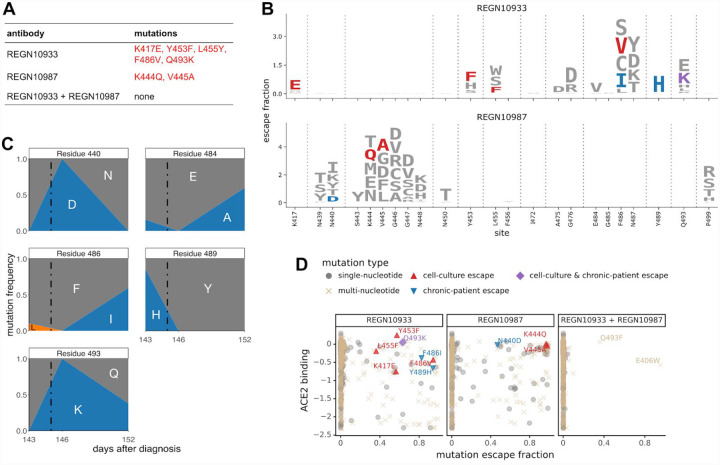
Escape maps are consistent with viral mutations selected in cell culture and a persistently infected patient. (A) Viral escape mutations selected by Regeneron with spike-pseudotyped VSV in cell culture in the presence of antibody (10). (B) Escape maps like those in Fig. 1A but showing only mutations accessible by single-nucleotide changes to the Wuhan-Hu-1 sequence, with non-gray colors indicating mutations in cell culture (red), in the infected patient (blue), or both (purple). Fig. S5 shows these maps colored by how mutations affect ACE2 affinity or RBD expression. (C) Dynamics of RBD mutations in a patient treated with REGN-COV2 at day 145 of his infection (black dashed vertical line). E484A rose in frequency in linkage with F486I, but since E484A is not an escape mutation in our maps it is not shown in other panels. See also Fig. S4. (D) The escape mutations that arise in cell culture and the infected patient are single-nucleotide accessible and escape antibody binding without imposing a large cost on ACE2 affinity. Each point is a mutation with shape / color indicating whether it is accessible and selected during viral growth. Points further to the right on the x-axis indicate stronger escape from antibody binding; points further up on the y-axis indicate higher ACE2 affinity.

**Figure 3.**
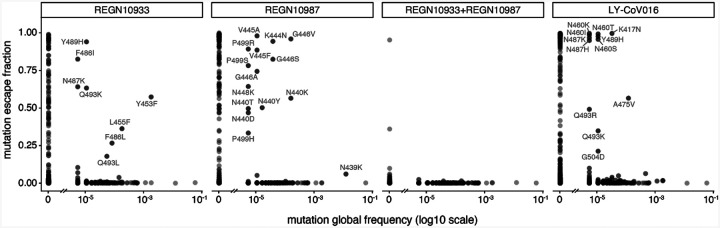
Antibody escape mutations in circulating SARS-CoV-2. For each antibody or antibody combination, the escape score for each mutation is plotted versus its frequency among the 180,555 high-quality human-derived SARS-CoV-2 sequences on GISAID (25) as of November 12, 2020. Escape mutations with notable GISAID frequencies are labeled.

**Figure 4.**
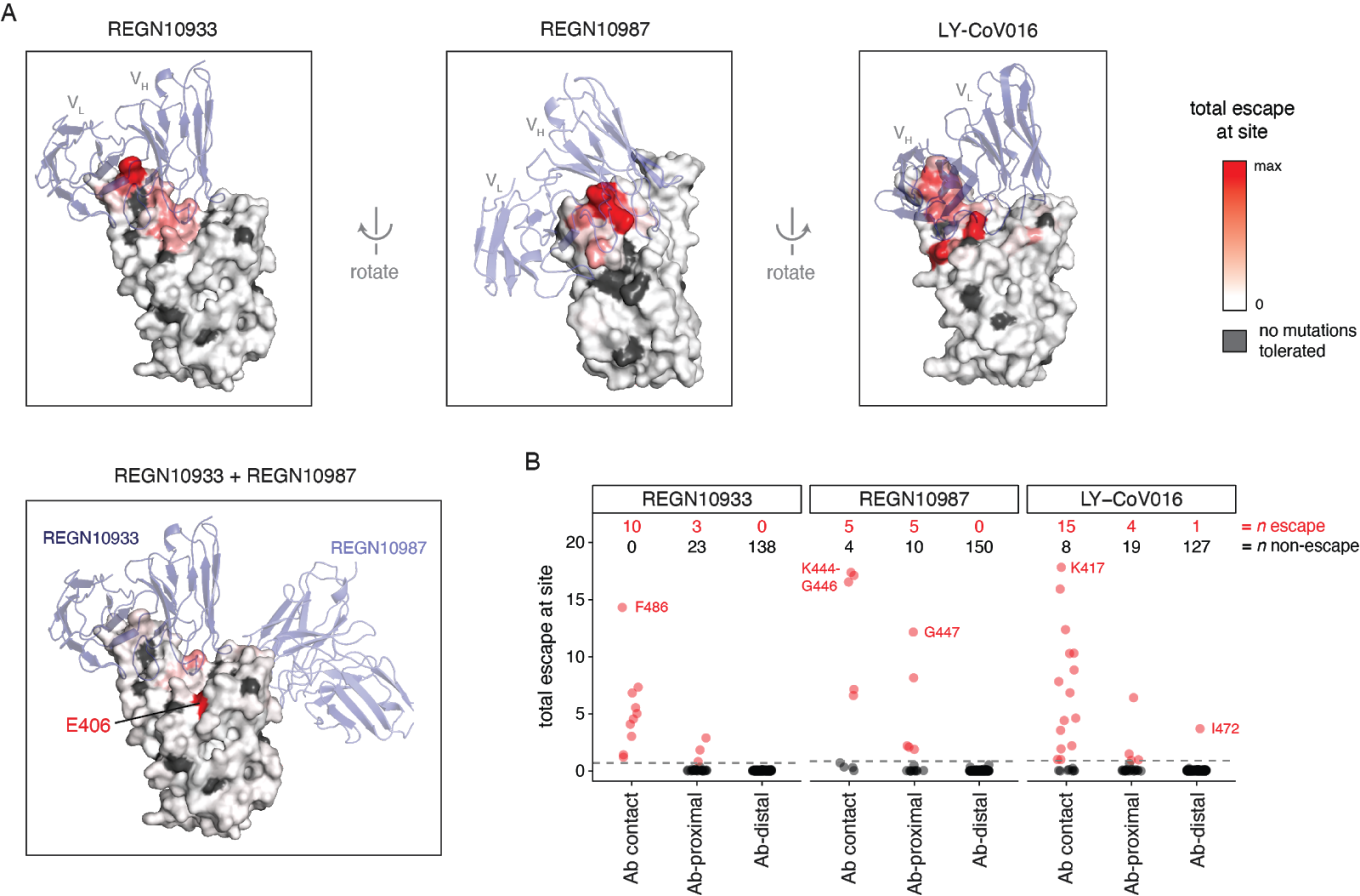
Structural context of escape mutations. (A) Escape maps projected on antibody-bound RBD structures. (REGN10933 and REGN10987: PDB 6XDG (9); LY-CoV016: PDB 7C01 (11)). Antibody heavy-and light-chain variable domains are shown as blue cartoons, and the RBD surface is colored to indicate how strongly mutations at that site mediate escape (white indicates no escape, red indicates strongest escape site for that antibody / cocktail). Sites where no mutations are functionally tolerated are colored gray. (B) For each antibody, sites were classified as direct antibody contacts (non-hydrogen atoms within 4Å of antibody), antibody-proximal (4–8Å), or antibody-distal (>8Å). Each point indicates a site, classified as escape (red) or non-escape (black) (dashed gray line, see Methods). Red and black numbers indicate how many sites in each category are escape or non-escape, respectively. Interactive visualizations are at https://jbloomlab.github.io/SARS-CoV-2-RBD_MAP_clinical_Abs/ and additional static views are in Fig. S6.
